# Comparing the Effect of DOAC-Stop^®^ and DOAC-Remove^®^ on Apixaban, Rivaroxaban and Dabigatran Prior to Thrombophilia and Lupus Testing

**DOI:** 10.3389/bjbs.2024.13359

**Published:** 2024-10-29

**Authors:** Noor-E.-Huddah Malik, Andrew Ward, Beth Erskine

**Affiliations:** ^1^ Department of Healthcare Science, University of Sunderland, Sunderland, United Kingdom; ^2^ Department of Haematology, Northumbria Healthcare NHS Foundation Trust, North Shields, United Kingdom

**Keywords:** DOAC-stop®, DOAC-remove®, DOAC-interference, thrombophilia testing, lupus anticoagulant testing

## Abstract

**Background:**

Direct oral anticoagulants (DOACs) interfere with coagulation assays potentially leading to inaccurate results. This study determined the effectiveness of DOAC-stop® and DOAC-remove® in overcoming DOAC interference. It aimed to investigate the extent to which apixaban, rivaroxaban, and dabigatran had an effect on thrombophilia and lupus tests using normal plasma, as well as whether DOACs interfere with true-positive results by testing abnormal controls.

**Methods:**

Apixaban (0.03 mg/mL), rivaroxaban (0.01 mg/mL), and dabigatran (0.019 mg/mL) stock solutions were made and added to the normal pool at three different concentrations (200, 400 and 600 ng/mL) and to the abnormal controls at a single concentration. These samples and untreated DOAC controls were tested before and after adding either DOAC-stop® or DOAC-remove®. The measured parameters included protein C, protein S, antithrombin III (ATIII), DRVVS, DRVVC, PTT-LA and DOAC concentration. The normal pool spiked with DOAC was repeated seven times for each DOAC at each concentration level and the abnormal controls spiked with DOAC were repeated four times at a single concentration level for each DOAC.

**Results:**

In the normal pool, dabigatran and rivaroxaban affected all lupus anticoagulant tests, whereas apixaban only affected DRVVS and DRVVC. While dabigatran led to false-positive protein S deficiency and falsely elevated ATIII. Both DOAC-stop® and DOAC-remove® brought the thrombophilia results and all falsely elevated lupus anticoagulant results back within the normal range for apixaban and rivaroxaban. For dabigatran all the affected lupus anticoagulant tests remained abnormal following DOAC-remove®, unlike DOAC-stop® treatment, where only DRVVS and DRVVC at 600 ng/mL remained abnormal. In abnormal controls, all DOACs falsely elevated the lupus anticoagulant tests, whereas dabigatran caused false negative ATIII results, that were corrected (remained abnormal) with DOAC-stop® and DOAC-remove®. DOAC-stop® showed a greater reduction in lupus anticoagulant results than DOAC-remove®, causing a false-negative DRVVT ratio for rivaroxaban.

**Conclusion:**

DOAC-stop® is more effective than DOAC-remove® in removing all DOACs below the reference range, whereas DOAC-remove® failed to remove dabigatran.

## Introduction

In the United Kingdom, approximately 1 in 20 people will have venous thromboembolism (VTE) at some point in their lives. The typical treatment for VTE, including pulmonary embolism (PE) and deep vein thrombosis (DVT), is predominantly comprised of vitamin K antagonists such as warfarin [1]. However, recent advances in treatment have included the introduction of direct oral anticoagulants (DOACs), as there is a lower risk of bleeding compared to warfarin, fewer complications related to VTE, reduced risk of recurrent VTE, less frequent coagulation monitoring and decreased interactions between the drug and food [2–5]. DOACs can be categorized into two main classes of DOACs: thrombin, factor II inhibitors, including dabigatran etexilate (Pradaxa®), or factor Xa inhibitors such as rivaroxaban (Xarelto®) and apixaban (Eliquis®). This ultimately prevents clot formation and indirectly impacts platelet aggregation [4]. DOACs have been approved to treat PE and DVT, prevent PE and DVT in patients who underwent hip replacement surgery and reduce the risk of stroke in patients with nonvalvular atrial fibrillation (NVAF) [4].

Hypercoagulability, or thrombophilia, is a condition that increases an individual’s likelihood of developing blood clots, leading to an increased risk of VTE [6]. Thrombophilia can be inherited or acquired [6]. In inherited thrombophilia, many mutations and genetic risk factors can contribute to a higher risk of thrombosis. As an example, a single nucleotide point mutation (SNP code: rs6025) in factor V results in Factor V Leiden (FVL), causing the protein C cleavage site to be eliminated and resulting in factor V remaining active, which increases the risk of thrombosis. Additionally, mutations that cause deficiencies in natural anticoagulants, such as ATIII, protein C, and protein S, can also lead to thrombophilia [7, 8]. Antiphospholipid syndrome, the presence of at least one antiphospholipid antibody, including Lupus anticoagulant, is one of the most common risk factors for acquired thrombophilia, leading to an increased chance of blood clot formation in the arteries and veins [9].

For this reason, following a VTE, it is crucial to assess the risk of thrombophilia to ensure that measures are taken to reduce the risk of complications, to identify the risk of recurring thrombotic events, and to determine suitable treatment options [8]. Therefore, a thrombophilia screen may be requested; however, prior to screening, the patient may be taking a DOAC to treat VTE and this may interfere with specific laboratory assays used for thrombophilia screening [10–13]. Possible solutions for patients who require thrombophilia screening but are taking DOACs include: temporarily stopping the anticoagulant medication but this may increase the risk of a thrombotic event; switching to low molecular weight heparin (which may interfere with Lupus anticoagulant tests); or using assays that are not sensitive to DOACs (but these assays may not be widely available and may be expensive to perform) [11]. For this reason, DOAC-stop® and DOAC-remove® are novel agents containing activated carbon that adsorb and remove DOACs from the patient sample without interfering with the plasma proteins involved in the clotting mechanism, allowing for thrombophilia testing [11].

Research has been conducted to investigate the effect of either DOAC-stop® or DOAC-remove® on various DOACs and assays. For example, studies by Baker et al. [14], De Kesel and Devreese [15], Favaloro et al. [16] and Ząbczyk et al. [17] all confirmed that DOAC-stop® effectively removes various DOACs and overcomes interference in Lupus anticoagulant testing [14–17]. Additionally, studies by Skaugen et al. [18], Monteyne et al. [19] and Jourdi et al. [20] found that DOAC-remove® was effective in reducing false-positive lupus anticoagulant results [18–20]. In particular, a study by Favresse et al. evaluated the use of DOAC-stop® to eliminate various DOACs for laboratory assays used for thrombophilia screening [21]. This study found that DOAC-stop® was effective in removing DOACs but the DOACs predominantly led to false-positive Lupus anticoagulant results such as Partial Thromboplastin Time - Lupus Anticoagulant (PTT-LA), Dilute Russell’s viper venom time (dRVVT) screen and dRVVT confirm, with variability depending on the assay used [21]. Additionally, this study did not determine the effect of DOACs on true-positive Lupus anticoagulant patients, patients with antithrombin, protein C, or protein S deficiency nor did it evaluate the effect of DOAC-remove®.

This project aims to compare the effectiveness of DOAC-stop® and DOAC-remove® in removing DOACs such as rivaroxaban, apixaban and dabigatran at various concentrations from normal pool plasma samples and abnormal controls so thrombophilia screening and lupus testing can be carried out. By addressing research gaps, this project could allow for more reliable testing, more accurate results and savings in time, money, and reagents. Additionally, this project will evaluate the effect of DOACs on specific laboratory assays such as the chromogenic protein C assay, factor-II-based antithrombin assay, the protein S antigen assay and clot-based PTT-LA and DRVVT assays.

## Methodology

### Spiking Normal Pool and Abnormal Controls

The normal human plasma pool (Pool Norm – REF 00539) was used as the standard reference. For abnormal controls, the abnormal controls for PTT-LA (STACoag Control P – REF 00679), ATIII and protein C (STA-System Control P – REF 00678), screen and confirm (STAControl LA 2 - REF 00201) and protein S (STALiatest Control P – REF 00526) were mixed together; it is acknowledged that pooling different abnormal controls does not guarantee a single abnormal control for all assays. All of the reagents were supplied by Diagnostica Stago UK Ltd. 2 Theale Lakes Business Park Moulden Way Theale RG7 4GB England. The normal pool and abnormal controls came in lyophilized form from the manufacturer and stored between 2°C and 8°C. Upon use, the normal pool and abnormal control vials were reconstituted with distilled water and kept at room temperature for 30 min before gently mixing (this was completed according to the manufacturer’s safety datasheet). Once reconstituted, according to the manufacturer’s datasheet, the normal pool and abnormal controls remained stable for 8 h at room temperature. Stock solutions of apixaban (0.03 mg/mL), rivaroxaban (0.01 mg/mL) and dabigatran (0.019 mg/mL) were prepared by adding apixaban (2.5 mg), rivaroxaban (10 mg) and dabigatran (110 mg) tablets to boiling water and stirring until the tablets dissolved; this protocol was adapted according to suggestions by Beth Erskine (personal communication). The DOAC tablets were provided by the local pharmacy. These stock solutions were added to 1.5 mL of the normal pool at three different volumes to obtain three different concentration levels of approximately 200 ng/mL, 400 ng/mL and 600 ng/mL. The stock solutions were also added to the mixed abnormal controls at a single concentration: the apixaban stock solution was used to achieve approximately 400 ng/mL, while rivaroxaban and dabigatran were used to achieve approximately 100 ng/mL each. DOAC-spiked and untreated samples without DOAC (either normal pool or abnormal controls) were tested before being treated with either DOAC-stop® or DOAC-remove®. For DOAC-spiked normal pool samples, each concentration level for each DOAC was repeated seven times for a total of 126 samples (not including four control samples for DOAC-stop® and eight control samples for DOAC-remove® without DOACs). For abnormal controls, there were four repeats for each DOAC, resulting in 24 samples at a single concentration level (not including two control samples without DOACs for both DOAC-stop® and DOAC-remove®). Ethical approval was not required for this project as it was a quality improvement study.

### DOAC-Stop® and DOAC-Remove® Procedure

After pre-testing, either DOAC-stop® (Haematex) or DOAC-remove® (5-Diagnostics) was added to the remaining sample. The sample was mixed for 10 min on a roller mixer. This was then centrifuged for 6 min at 4,400 rpm. The supernatant was pipetted carefully into a microtainer to avoid pipetting the pellet and tested again. This was completed according to the manufacturer’s instructions.

### Coagulation Testing

All tests were performed on the STA-R MAX 2 analyzer (Diagnostica Stago) and appropriate calibration and quality controls were performed to ensure the accuracy of the results. For thrombophilia testing, the protein S (STA-Liatest Free Protein S) was tested using an antigenic assay, protein C (STA-Stachrom Protein C) was tested using a chromogenic assay and the ATIII activity (STA-Stachrom AT III) was tested using a thrombin-based assay. For lupus anticoagulant testing, the clot-based assays, dRVVT screen and confirm (STA-Staclot dRVV Screen and Confirm) and PTT-LA were performed. A screen-to-confirm ratio was calculated using the results from the dRVVT screen and confirm. For apixaban and rivaroxaban, concentrations were measured using a chromogenic assay (STA-liquid anti-Xa). However, dabigatran concentrations were measured using an ecarin chromogenic assay (STA-ECA-II).

### Statistical Analysis

The IBM SPSS statistics software (version 29.0.1.0) was used to perform all statistical analyses. Statistical comparisons were carried out using the Mann-Whitney U test. A P-value of less than 0.05 (P < 0.05) was considered statistically significant.

## Results

### Normal Pool: DOAC-Remove® and DOAC-Stop®

Tables 1, 2 (pre rows) show the various parameters that are directly affected by the DOACs. Tables 1, 2 show that apixaban and rivaroxaban did not affect the thrombophilia screening tests (protein C, protein S and ATIII). Although some parameters differ significantly from the control, all mean values are within the normal range. For dabigatran, at 400 ng/mL and 600 ng/mL, the ATIII concentration was falsely elevated above the reference range. However, this is not clinically significant. Additionally, when the dabigatran concentration exceeded 400 ng/mL (as seen in Table 1) or 600 ng/mL (as seen in both Tables 1, 2), then the protein S concentration decreased below the reference range resulting in a false-positive protein S deficiency. In contrast to the thrombophilia screening tests, all DOACs interfered with the lupus anticoagulant tests, causing false-positive results. Rivaroxaban and dabigatran both affected all of the lupus anticoagulant tests (DRVVS, DRVVC, DRVVT ratio and PTT-LA) at all concentrations, as reflected by false elevations of all tests above the reference range. However, apixaban only affected the DRVVS and DRVVC, resulting in elevated results, but from these two values the DRVVT ratio subsequently stayed within the normal range. Moreover, PTT-LA was unaffected by apixaban at all concentrations.

**TABLE 1:** DOAC-remove® – Mean values of the various parameters before and after the addition of DOAC-remove® to normal pool at the specified concentration (pre/post comparison).

		DOAC Remove									
		Control pool norm (n = 16)	Dabigatran			Apixaban			Rivaroxaban		
			200 ng/mL (n = 14)	400 ng/mL (n = 14)	600 ng/mL (n = 14)	200 ng/mL (n = 14)	400 ng/mL (n = 14)	600 ng/mL (n = 14)	200 ng/mL (n = 14)	400 ng/mL (n = 14)	600 ng/mL (n = 14)
Mean Protein C (IU/dL) normal range: 70–130 IU/dL	Pre	105.6 (11.0)	93.9 (1.1)	112.0 (7.2)	95.7 (4.3)	117.4 (2.5)	95.4 (0.5)	95.0 (0.6)	119.7 (4.9)	104.4 (2.1)	94.6 (2.5)
	Post	106.9 (12.9)	95.6 (1.9)	116.6 (2.5)	96.7 (4.3)	116.6 (2.8)	93.0 (1.2)	100.7 (5.0)	121.3 (2.8)	105.1 (1.1)	87.7 (1.1)
	P-value	**P = 0.798**	**P = 0.097**	**P = 0.165**	**P = 0.259**	**P = 0.535**	**P < 0.001**	**P = 0.165**	**P = 0.318**	**P = 0.383**	**P < 0.001**
Mean Protein S (IU/dL) normal range: 70–130 IU/dL	Pre	91.6 (3.3)	83.6 (2.7)	68.1 (3.2)	60.3 (6.8)	90.0 (2.2)	89.7 (3.3)	89.6 (3.5)	89.9 (1.4)	87.1 (2.3)	86.6 (1.7)
	Post	90.1 (2.0)	89.3 (1.0)	82.7 (3.5)	78.9 (3.1)	90.0 (2.9)	85.7 (0.6)	87.9 (2.2)	87.9 (1.9)	85.6 (1.3)	87.9 (3.1)
	P-value	**P = 0.328**	**P < 0.001**	**P < 0.001**	**P < 0.001**	**P = 0.805**	**P = 0.017**	**P = 0.383**	**P = 0.073**	**P = 0.128**	**P = 0.535**
Mean Antithrombin III (IU/dL) normal range: 80–120 IU/dL	Pre	102.5 (6.1)	118.3 (1.4)	121.9 (4.7)	124.3 (2.0)	110.6 (3.4)	99.3 (2.7)	98.9 (3.9)	98.9 (1.9)	98.6 (1.1)	97.3 (2.2)
	Post	102.6 (6.2)	107.0 (2.4)	107.3 (2.4)	107.4 (5.3)	107.9 (3.6)	98.6 (2.7)	101.7 (2.6)	96.6 (1.4)	99.4 (1.5)	100.6 (3.2)
	P-value	**P = 0.878**	**P < 0.001**	**P < 0.001**	**P < 0.001**	**P = 0.165**	**P = 0.456**	**P= 0.209**	**P = 0.053**	**P = 0.535**	**P = 0.017**
Mean DRVVS (ratio) normal range: <1.2	Pre	1.0 (0.04)	3.1 (0.2)	4.4 (0.2)	4.5 (0.3)	1.5 (0.1)	1.8 (0.1)	2.0 (0.2)	2.4 (0.2)	3.1 (0.1)	2.8 (0.3)
	Post	1.0 (0.1)	1.6 (0.1)	2.3 (0.2)	2.2 (0.2)	1.0 (0.01)	1.0 (0.02)	1.0 (0.02)	1.1 (0.1)	1.1 (0.03)	1.0 (0.01)
	P-value	**P = 0.798**	**P < 0.001**	**P < 0.001**	**P < 0.001**	**P < 0.001**	**P < 0.001**	**P < 0.001**	**P < 0.001**	**P < 0.001**	**P < 0.001**
Mean DRVVC (ratio) normal range: <1.2	Pre	1.0 (0.03)	2.5 (0.1)	3.2 (0.1)	3.5 (0.1)	1.7 (0.1)	2.0 (0.1)	2.2 (0.2)	1.8 (0.1)	2.1 (0.04)	2.0 (0.2)
	Post	1.0 (0.04)	1.5 (0.04)	1.8 (0.1)	1.8 (0.1)	1.0 (0.02)	1.0 (0.02)	1.0 (0.01)	1.0 (0.01)	1.0 (0.03)	1.0 (0.02)
	P-value	**P = 279**	**P < 0.001**	**P < 0.001**	**P < 0.001**	**P < 0.001**	**P < 0.001**	**P < 0.001**	**P < 0.001**	**P < 0.001**	**P < 0.001**
Mean DRVVT Ratio normal range: <1.2	Pre	1.0 (0.03)	1.2 (0.01)	1.4 (0.1)	1.3 (0.04)	0.9 (0.03)	0.9 (0.01)	0.9 (0.02)	1.4 (0.1)	1.5 (0.04)	1.4 (0.1)
	Post	1.0 (0.04)	1.1 (0.01)	1.3 (0.02)	1.2 (0.04)	1.0 (0.02)	1.0 (0.02)	1.0 (0.01)	1.1 (0.1)	1.1 (0.04)	1.0 (0.02)
	P-value	**P = 0.105**	**P < 0.001**	**P = 0.004**	**P = 0.004**	**P < 0.001**	**P < 0.001**	**P < 0.001**	**P < 0.001**	**P < 0.001**	**P < 0.001**
Mean PTT-LA (seconds) normal range: 34–41 s	Pre	34.5 (2.2)	82.0 (6.2)	114.1 (6.6)	125.7 (2.1)	38.6 (1.5)	38.3 (0.3)	39.1 (0.9)	39.9 (0.6)	46.3 (0.8)	46.0 (2.2)
	Post	34.4 (2.1)	47.9 (1.9)	61.8 (4.8)	60.2 (3.7)	34.6 (0.8)	33.8 (0.6)	33.7 (0.8)	38.7 (0.6)	34.8 (1.1)	33.3 (0.3)
	P-value	**P = 1**	**P < 0.001**	**P < 0.001**	**P < 0.001**	**P < 0.001**	**P < 0.001**	**P < 0.001**	**P = 0.002**	**P < 0.001**	**P < 0.001**
Mean DOAC concentration (ng/mL): <30 ng/mL	Pre	0	255.1 (49.6)	452.4 (6.2)	**M < Mmin** ^#^	197.3 (27.4)	402.6 (20.0)	564.9 (33.6)	161.6 (35.8)	371.4 (30.6)	562.9 (29.3)
	Post	0	45.7 (15.3)	72.1 (20.3)	82.7 (29.1)	0.9 (1.1)	8.6 (4.5)	4.9 (3.6)	1.3 (1.7)	4.9 (4.3)	3.3 (1.8)
	P-value	—	**P < 0.001**	**P < 0.001**	**N.A**	**P < 0.001**	**P < 0.001**	**P < 0.001**	**P < 0.001**	**P < 0.001**	**P < 0.001**

Notes: The mean (1 standard deviation) of each parameter is presented.

# - unable to obtain a value as the result exceeded the limit of quantification.

If the *p*-value is black this indicates that the mean is within the normal range, whether or not there is a significant difference.

If the *p*-value is red this indicates that there was a significant difference and that abnormal means were brought within the normal range.

If the *p*-value is blue this indicates that there was a significant difference and abnormal results remained abnormal which is clinically significant.

**TABLE 2:** DOAC-Stop® – Mean values of the various parameters before and after the addition of DOAC-stop® to normal pool at the specified concentration (pre/post comparison).

		DOAC-Stop									
		Control pool norm (n = 8)	Dabigatran			Apixaban			Rivaroxaban		
			200 ng/mL (n = 14)	400 ng/mL (n = 14)	600 ng/mL (n = 14)	200 ng/mL (n = 14)	400 ng/mL (n = 14)	600 ng/mL (n = 14)	200 ng/mL (n = 14)	400 ng/mL (n = 14)	600 ng/mL (n = 14)
Mean Protein C (IU/dL) normal range: 70–130 IU/dL	Pre	104.5 (1.7)	95.6 (1.9)	101.0 (2.0)	99.9 (0.7)	101.0 (1.6)	101.3 (0.8)	106.4 (2.4)	105.1 (3.3)	103.0 (2.3)	101.1 (4.8)
	Post	103.0 (2.7)	95.1 (1.9)	98.9 (1.0)	103.3 (6.8)	101.1 (1.1)	100.0 (1.2)	111.4 (3.6)	108.0 (2.8)	101.9 (3.0)	100.7 (3.7)
	P-value	**P = 0.486**	**P = 0.209**	**P = 0.053**	**P = 0.805**	**P = 0.209**	**P = 0.053**	**P = 0.011**	**P = 0.073**	**P = 0.259**	**P = 1**
Mean Protein S (IU/dL) normal range: 70–130 IU/dL	Pre	93.8 (2.1)	83.6 (1.9)	72.3 (5.0)	65.7 (2.7)	90.1 (2.0)	89.1 (2.9)	96.1 (3.1)	91.3 (0.8)	89.0 (1.2)	89.6 (2.0)
	Post	92.0 (1.9)	89.6 (2.2)	89.0 (3.9)	86.7 (1.7)	88.0 (1.4)	87.6 (1.3)	92.7 (1.9)	89.4 (1.1)	89.7 (1.1)	89.7 (1.4)
	P-value	**P = 0.343**	**P < 0.001**	**P < 0.001**	**P < 0.001**	**P = 0.038**	**P = 0.259**	**P = 0.017**	**P = 0.011**	**P = 0.318**	**P = 0.805**
Mean Antithrombin III (IU/dL) normal range: 80–120 IU/dL	Pre	103.8 (1.7)	115.7 (2.4)	122.7 (2.6)	129.7 (2.0)	103.0 (1.8)	102.3 (2.6)	109.6 (2.6)	107.1 (2.5)	102.4 (2.2)	98.6 (3.6)
	Post	103.5 (1.9)	105.1 (3.7)	100.6 (3.2)	98.4 (2.8)	102.9 (1.6)	102.3 (2.9)	109.0 (3.4)	105.4 (3.0)	101.6 (2.3)	100.0 (3.0)
	P-value	**P = 0.886**	**P < 0.001**	**P < 0.001**	**P < 0.001**	**P = 1**	**P = 0.902**	**P = 0.805**	**P = 0.318**	**P = 0.456**	**P = 0.456**
Mean DRVVS (ratio) normal range: <1.2	Pre	1.0 (0.1)	3.1 (0.1)	4.0 (0.1)	4.6 (0.1)	1.6 (0.1)	1.9 (0.1)	2.4 (0.1)	2.5 (0.1)	2.7 (0.3)	2.7 (0.2)
	Post	1.0 (0.1)	1.1 (0.03)	1.2 (0.1)	1.3 (0.1)	1.0 (0.02)	1.0 (0.02)	1.1 (0.01)	1.1 (0.1)	1.0 (0.02)	1.0 (0.03)
	P-value	**P = 0.686**	**P < 0.001**	**P < 0.001**	**P < 0.001**	**P < 0.001**	**P < 0.001**	**P < 0.001**	**P < 0.001**	**P < 0.001**	**P < 0.001**
Mean DRVVC (ratio) normal range: <1.2	Pre	1.0 (0.1)	2.5 (0.1)	3.1 (0.1)	3.7 (0.1)	1.6 (0.1)	2.1 (0.1)	2.4 (0.2)	1.8 (0.1)	2.0 (0.1)	2.0 (0.1)
	Post	1.0 (0.01)	1.1 (0.02)	1.1 (0.03)	1.3 (0.1)	1.0 (0.01)	1.0 (0.03)	1.0 (0.01)	1.0 (0.01)	1.0 (0.02)	1.0 (0.02)
	P-value	**P = 0.886**	**P < 0.001**	**P < 0.001**	**P < 0.001**	**P < 0.001**	**P < 0.001**	**P < 0.001**	**P < 0.001**	**P < 0.001**	**P < 0.001**
Mean DRVVT Ratio normal range: <1.2	Pre	1.0 (0.1)	1.2 (0.02)	1.3 (0.1)	1.2 (0.03)	1.0 (0.03)	0.9 (0.03)	1.0 (0.1)	1.4 (0.03)	1.3 (0.1)	1.3 (0.1)
	Post	TABLE 2	1.0 (0.02)	1.0 (0.03)	1.0 (0.03)	1.0 (0.01)	1.0 (0.04)	1.1 (0.02)	1.1 (0.1)	1.0 (0.01)	1.0 (0.01)
	P-value	**P = 1**	**P < 0.001**	**P < 0.001**	**P < 0.001**	**P = 0.038**	**P = 0.001**	**P < 0.001**	**P < 0.001**	**P < 0.001**	**P < 0.001**
Mean PTT-LA (seconds) normal range: 34–41 s	Pre	32.4 (0.6)	76.8 (2.3)	103.1 (2.9)	123.6 (3.6)	36.1 (0.8)	38.2 (0.4)	40.4 (0.7)	42.6 (0.8)	47.7 (1.8)	46.7 (2.4)
	Post	32.0 (0.6)	34.6 (0.6)	36.0 (1.0)	40.1 (1.9)	32.0 (0.5)	32.4 (0.4)	32.6 (0.4)	33.1 (0.3)	33.9 (0.7)	33.5 (0.4)
	P-value	**P = 0.486**	**P < 0.001**	**P < 0.001**	**P < 0.001**	**P < 0.001**	**P < 0.001**	**P < 0.001**	**P < 0.001**	**P < 0.001**	**P < 0.001**
Mean DOAC concentration (ng/mL): <30 ng/mL	Pre	0	200.0 (17.0)	465.3 (11.8)	**M < Mmin** ^#^	236.4 (33.5)	449.4 (36.0)	576.1 (25.0)	184.7 (28.3)	440.9 (34.7)	555.1 (34.6)
	Post	0	2.0 (1.5)	5.4 (2.5)	10.1 (5.1)	5.0 (3.1)	6.7 (3.3)	6.6 (1.1)	9.4 (8.1)	7.7 (8.5)	7.7 (7.7)
	P-value	—	**P < 0.001**	**P < 0.001**	**N.A**	**P < 0.001**	**P < 0.001**	**P < 0.001**	**P < 0.001**	**P < 0.001**	**P < 0.001**

Notes: The mean (1 standard deviation) of each parameter is presented.

# - unable to obtain a value as the result exceeded the limit of quantification.

If the *p*-value is black this indicates that the mean is within the normal range, whether or not there is a significant difference.

If the *p*-value is red this indicates that there was a significant difference and abnormal means were brought within the normal range.

If the *p*-value is blue this indicates that there was a significant difference and abnormal results remained abnormal which is clinically significant.

If the *p*-value is orange this indicates that there was a significant difference and the result is in the abnormal range but is not clinically significant.

According to Table 1, DOAC-remove® successfully removed an average of 98.9% of apixaban, 99.1% of rivaroxaban and 85.6% of dabigatran across the three concentrations. In comparison, as shown in Table 2, DOAC-stop® successfully removed an average of 98.4% of apixaban, 97.3% of rivaroxaban and 98.9% of dabigatran across the three concentrations. This can be seen in Tables 1, 2, which show that DOAC-remove® is only able to remove apixaban and rivaroxaban to below 30 ng/mL (where the DOAC has a negligible effect) but not dabigatran; however, DOAC-stop® can remove all three DOACs effectively below 30 ng/mL.

Tables 1, 2 (post rows) show the impact of DOAC-remove® and DOAC-stop®, respectively. Both DOAC-Remove® and DOAC-Stop® successfully returned the falsely elevated DRVVS and DRVVC values caused by apixaban and rivaroxaban, as well as the DRVVT ratio and PTT-LA affected by rivaroxaban, to within the normal range. As shown in Table 2, the mean values of PTT-LA for all apixaban concentrations and 200 ng/mL for rivaroxaban decreased below the reference range after the addition of DOAC-stop®, but this is not clinically significant. The mean values of the other results in the apixaban and rivaroxaban groups remained within the normal range, although some parameters were significantly different from the control.

As shown in Tables 1, 2 for dabigatran, the protein S and ATIII concentrations were returned to the normal range with both DOAC-remove® and DOAC-stop®. This is not the case for the lupus anticoagulant tests; as with DOAC-remove® (as shown in Table 1), the DRVVS, DRVVC, and DRVVT ratios (except 200 ng/mL) and PTT-LA remained falsely elevated following treatment with DOAC-remove®. Table 2 shows that DOAC-stop® had a better outcome for dabigatran as only DRVVS and DRVVC at 600 ng/mL remained falsely elevated within the abnormal range. This can be explained by the fact that DOAC-stop® removed an average of 13.3% more dabigatran than DOAC-remove®.

No significant difference was found between the control parameters before and after the addition of either DOAC-remove® or DOAC-stop®.

### Abnormal Controls – DOAC-Remove® and DOAC-Stop®

As shown in Tables 3, 4 (pre rows), the results suggest that apixaban and rivaroxaban did not affect the thrombophilia tests (protein C, protein S and ATIII) as the mean values of these parameters with the addition of the DOAC are related to the controls without the DOAC and remained within the abnormal range. On the other hand, dabigatran did not affect protein C and protein S concentrations but caused the ATIII concentration to become falsely elevated, bringing it within the normal range and resulting in a false-negative result (93 IU/dL/90 IU/dL, respectively.) All three DOACs further elevated all the lupus anticoagulant tests (DRVVC, DRVVS and PTT-LA), resulting in more abnormal values. Additionally, rivaroxaban caused an elevation of the DRVVT ratio; however, dabigatran and apixaban did not affect the DRVVT ratio as they remained below the control value without the DOAC. However, the small sample size precludes drawing definitive conclusions and these findings should be interpreted with caution.

**TABLE 3:** Mean values of the various parameters before and after the addition of DOAC-remove^®^ to the spiked abnormal controls at a single concentration (pre/post comparison).

		DOAC-remove					
		Control (n = 2) (Abnormal control without DOAC)	Dabigatran (n = 8)	Control (n = 2) (Abnormal control without DOAC)	Apixaban (n = 8)	Control (n = 2) (Abnormal control without DOAC)	Rivaroxaban (n = 8)
Mean Protein C (IU/dL) normal range: 70–130 IU/dL	Pre	64	62.3 (0.5)	57	60.0 (2.7)	64	64.3 (1.9)
	Post	63	63.0 (0.8)	58	60.3 (3.2)	63	63.0 (1.8)
	P-value	—	**P = 0.200**	—	**P = 0.886**	—	**P = 0.486**
Mean Protein S (IU/dL) normal range: 70–130 IU/dL	Pre	55	54.0 (0.8)	52	53.8 (1.7)	55	55.5 (1.3)
	Post	54	52.8 (1.7)	50	51.3 (1.0)	54	55.3 (1.9)
	P-value	—	**P = 0.343**	—	**P = 0.057**	—	**P = 0.686**
Mean Antithrombin III (IU/dL) normal range: 80–120 IU/dL	Pre	72	93.3 (4.8)	55	58.8 (2.1)	72	69.8 (3.0)
	Post	74	73.0 (3.6)	56	60.0 (2.7)	74	69.0 (1.8)
	P-value	—	**P = 0.029**	—	**P = 0.686**	—	**P = 0.886**
Mean DRVVS (ratio) normal range: <1.2	Pre	2.32	5.4 (0.3)	2.41	5.2 (0.1)	2.32	6.2 (0.2)
	Post	2.26	2.4 (0.04)	2.32	2.4 (0.01)	2.26	2.1 (0.1)
	P-value	—	**P = 0.029**	—	**P = 0.029**	—	**P = 0.029**
Mean DRVVC (ratio) normal range: <1.2	Pre	1.44	3.7 (0.3)	1.54	4.1 (0.03)	1.44	2.6 (0.1)
	Post	1.47	1.6 (0.03)	1.52	1.5 (0.04)	1.47	1.6 (0.01)
	P-value	—	**P = 0.029**	—	**P = 0.029**	—	**P = 0.029**
Mean DRVVT Ratio normal range: <1.2	Pre	1.61	1.5 (0.02)	1.56	1.3 (0.03)	1.61	2.4 (0.1)
	Post	1.54	1.5 (0.03)	1.53	1.5 (0.03)	1.54	1.3 (0.1)
	P-value	—	**P = 0.200**	—	**P = 0.200**	—	**P = 0.200**
Mean PTT-LA (seconds) normal range: 34–41 s	Pre	76.7	170.1 (10.7)	82.3	137.0 (3.5)	76.7	104.8 (0.8)
	Post	73.5	80.5 (1.0)	78.6	77.5 (0.7)	73.5	70.6 (0.3)
	P-value	—	**P = 0.029**	—	**P = 0.029**	—	**P = 0.029**
Mean DOAC concentration (ng/mL): <30 ng/mL	Pre	0	158.3 (32.6)	0	373.8 (15.3)	0	58.3 (1.0)
	Post	0	6.3 (0.5)	0	5.0 (4.7)	0	6.3 (1.7)
	P-value	—	**P = 0.029**	—	**P = 0.029**	—	**P = 0.029**

Notes: The mean (1 standard deviation) of each parameter is presented.

If the *p*-value is black this indicates that the mean is within the abnormal range, whether or not there is a significant difference.

If the *p*-value is red this indicates that there was a significant difference and normal means were brought within the abnormal range.

If the *p*-value is orange this indicates that there was a significant difference and the result has been brought within the normal range.

If the mean is highlighted red this indicates that it is within normal range (should be abnormal), which is clinically significant.

**TABLE 4:** Mean values of the various parameters before and after the addition of DOAC-stop^®^ to the spiked abnormal controls at a single concentration (pre/post comparison).

		DOAC-Stop					
		Control (n = 2) (Abnormal control without DOAC)	Dabigatran (n = 4)	Control (n = 2) (Abnormal control without DOAC)	Apixaban (n = 4)	Control (n = 2) (Abnormal control without DOAC)	Rivaroxaban (n = 4)
Mean Protein C (IU/dL) normal range: 70–130 IU/dL	Pre	62	62.0 (0.0)	58	59.0 (0.8)	62	62.3 (1.5)
	Post	62	61.5 (0.6)	57	57.8 (2.1)	62	62.0 (0.8)
	P-value	—	**P = 0.343**	—	**P = 0.486**	—	**P = 0.886**
Mean Protein S (IU/dL) normal range: 70–130 IU/dL	Pre	55	54.3 (0.5)	55	52.8 (2.2)	55	54.3 (1.3)
	Post	54	53.0 (0.8)	54	51.8 (2.9)	54	52.5 (0.6)
	P-value	—	**P = 0.057**	—	**P = 0.686**	—	**P = 0.057**
Mean Antithrombin III (IU/dL) normal range: 80–120 IU/dL	Pre	72	90.0 (1.8)	60	56.8 (2.2)	72	71.5 (0.6)
	Post	68	69.0 (0.8)	58	53.8 (2.9)	68	69.5 (1.9)
	P-value	—	**P = 0.029**	—	**P = 0.114**	—	**P = 0.114**
Mean DRVVS (ratio) normal range: <1.2	Pre	2.24	4.8 (0.1)	2.36	5.4 (0.2)	2.24	6.3 (0.2)
	Post	1.6	1.7 (0.03)	1.73	2.5 (0.2)	1.6	1.5 (0.02)
	P-value	—	**P = 0.029**	—	**P = 0.029**	—	**P = 0.029**
Mean DRVVC (ratio) normal range: <1.2	Pre	1.45	3.2 (0.03)	1.69	4.0 (0.1)	1.45	2.6 (0.2)
	Post	1.26	1.3 (0.01)	1.5	1.5 (0.1)	1.26	1.4 (0.03)
	P-value	—	**P = 0.029**	—	**P = 0.029**	—	**P = 0.029**
Mean DRVVT Ratio normal range: <1.2	Pre	1.54	1.5 (0.02)	1.4	1.3 (0.1)	1.54	2.4 (0.2)
	Post	1.27	1.3 (0.03)	1.15	1.7 (0.2)	1.27	1.1 (0.02)
	P-value	—	**P = 0.029**	—	**P = 0.029**	—	**P = 0.029**
Mean PTT-LA (seconds) normal range: 34–41 s	Pre	74.4	150.7 (2.7)	76.3	130.1 (1.1)	74.4	106.3 (1.1)
	Post	56.1	55.7 (0.2)	55.3	76.6 (2.8)	56.1	54.6 (0.6)
	P-value	—	**P = 0.029**	—	**P = 0.029**	—	**P = 0.029**
Mean DOAC concentration (ng/mL): <30 ng/mL	Pre	0	117.0 (4.2)	0	366.3 (12.5)	0	63.3 (6.8)
	Post	0	1.3 (1.3)	0	2.3 (2.2)	0	6.0 (1.6)
	P-value	—	**P = 0.029**	—	**P = 0.029**	—	**P = 0.029**

Notes: The mean (1 standard deviation) of each parameter is presented.

If the *p*-value is black this indicates that the mean is within the abnormal range, whether or not there is a significant difference.

If the *p*-value is red this indicates that there was a significant difference and normal means were brought within the abnormal range.

If the *p*-value is orange this indicates that there was a significant difference and the result has been brought within the normal range.

If the mean is highlighted red this indicates that it is within normal range (should be abnormal), which is clinically significant.

As shown in Tables 3, 4 (post rows), DOAC-remove® and DOAC-stop® did not interfere with any thrombophilia results except ATIII for dabigatran, which returned to the abnormal range. From Table 3 for DOAC-remove® across all three DOACS, the falsely elevated lupus anticoagulant results (DRVVC, DRVVS and PTT-LA) were decreased (remaining within the abnormal range) and stayed in conjunction with the results from pre-treatment and control values. For dabigatran and apixaban, the DRVVT ratio increased and for rivaroxaban it decreased after post-testing with DOAC-remove®, which was similar to the abnormal controls without the DOAC. The abnormal controls without DOAC remained persistent before and after the addition of DOAC-remove® for all parameters.

As shown in Table 4, according to the controls, the results following DOAC-stop® decreased more in comparison to the controls from pre-testing only for the lupus anticoagulant tests. All control values remained within the abnormal range, except the DRVVT ratio for apixaban, which decreased to within the normal range. For all three DOACs following testing with DOAC-stop®, the falsely elevated DRVVS, DRVVC and PTT-LA were reduced but remained abnormal. However, for apixaban, the PTT-LA value following DOAC-stop® remained associated with the control from pre-testing, in contrast to the PTT-LA values for dabigatran and rivaroxaban which were similar to the controls from post-testing. DOAC-stop has also appeared to reduce the PTT-LA value for dabigatran (63%) more than for apixaban (41%) and rivaroxaban (48%). For both dabigatran and rivaroxaban, the DRVVT ratio decreased; however, for rivaroxaban, it decreased to be within the normal range, resulting in a false-negative value. For apixaban, the DRVVT ratio increased from pre-testing and the value was associated with the control from pre-testing rather than post-testing.

Given the small sample size, these observations should be viewed as preliminary, with further research necessary to validate these findings.

In the abnormal controls, DOAC-stop® removed 98.9% of dabigatran, 99.4% of apixaban and 90.5% of rivaroxaban. DOAC-remove® removed 96.1%, 98.7% and 89.3% of dabigatran, apixaban and rivaroxaban, respectively.

## Discussion

The DRVVT ratio and PTT-LA laboratory results will be prolonged if the patient has a lupus anticoagulant. If the patient has a deficiency in either protein C, protein S or ATIII, this will demonstrate that the patient has thrombophilia. DOACs have become an increasingly popular treatment for VTE. This study has shown that while DOACs do not interfere with every assay in this study, they do have an effect on some, resulting in false-positive or false-negative results that are detrimental to the patient. Tables 1–4 show that apixaban and rivaroxaban did not affect any of the thrombophilia assays (protein C, protein S and ATIII) and many studies support this idea [11, 21–24]. These results may be explained by the underlying type of assay used to measure these proteins. For example, protein C is measured by a chromogenic assay that directly measures the amount of protein C in the sample via a chromogenic substrate that binds to protein C and the measured optical density is proportional to the amount of protein C in the sample [25]. Furthermore, protein S is measured using an antigenic (immuno-turbidimetric) assay, which involves using antibodies that are covalently bound to latex particles; this causes agglutination in the presence of protein S (which increases turbidity); therefore, light absorbance is proportional to the amount of protein S present in the sample [26]. Finally, the ATIII is measured by a factor II-based chromogenic assay. This assay relies on endogenous ATIII to inhibit thrombin and any thrombin present will cleave a chromogenic substrate so the absorbance can be measured, which is inversely proportional to ATIII [27]. Studies by Gosselin et al. and Mani both stated that protein C and protein S would be overestimated in the presence of rivaroxaban and apixaban if using clotting assays as clotting assays rely on the functional activity of the protein and DOACs interfere with the clotting cascade, prolonging clotting times, unlike the chromogenic and antigenic assays which detect the quantity present [23, 24]. These studies also stated that ATIII would be overestimated in the presence of Xa inhibitors if measured using the factor Xa assay (as opposed to the factor II assay), as this assay relies on ATIII to inhibit factor Xa, so Xa would be falsely decreased resulting in an overestimation of ATIII [23, 24].

Tables 1, 2 show that high concentrations of dabigatran (above 400 ng/mL) cause both falsely elevated ATIII results and falsely decreased protein S results (p < 0.05). Many articles state that ATIII is elevated in the presence of dabigatran when using a factor II-based assay [13, 21, 23, 24, 28–30]. This is because dabigatran is a thrombin inhibitor and therefore competes with ATIII, resulting in reduced thrombin in the sample and a low absorbance, which causes overestimated antithrombin (as they are inversely proportional). However, many studies state that dabigatran (or any DOAC) has no effect on the antigenic protein S assay, but in this study, protein S was decreased with increasing levels of dabigatran [13, 23, 24, 28, 29]. There may be several reasons for this; for example, dabigatran may indirectly decrease protein S activity by directly affecting thrombin (but if this were the case, protein C would be decreased, which was not seen), it may directly interfere with protein S which could interfere with agglutination or it may interfere with the stability of the assay. However, more research must be conducted to understand why this occurred. As seen in Tables 3, 4, the abnormal controls showed that the ATIII was elevated into the normal range in the presence of dabigatran, resulting in a false-negative result and a failure to detect the underlying deficiency. An article by Lindahl et al. supports this, stating that patients with ATIII deficiency will have false-negative results in the presence of dabigatran and thrombophilia may be falsely excluded [31].

The basis for *in vitro* detection of the lupus anticoagulant involves a prolongation of clotting times. In the DRVVT assay, if the lupus anticoagulant is present, antiphospholipid antibodies will bind to phospholipid components, causing a decreased activity for the prothrombinase complex as its ability to bind to the phospholipid surface is reduced ultimately reducing thrombin generation and prolonging clotting times [32]. Additionally, the PTT-LA please test is based on the principle that the lupus anticoagulant binds to the phospholipids that are used as one of the reagents in the APTT test, causing an abnormally prolonged clotting time and this test is sensitive to lupus anticoagulants [32]. Tables 1, 2 show that dabigatran and rivaroxaban falsely elevate all lupus anticoagulant tests (DRVVS, DRVVC, DRVVT ratios and PTT-LA please), whereas apixaban only affects DRVVS and DRVVC, resulting in false positives. Many articles confirm that DOACS affect the lupus anticoagulant tests [12, 14, 17, 20, 21, 23, 24, 33]. DOACs impact clotting tests like DRVVT and PTT-LA please by interfering with thrombin generation and the conversion of fibrinogen to fibrin, thereby prolonging clotting times. With apixaban, the DRVVS and DRVVC are out of range; however, the ratio is within the normal range, as seen in a study by Kovač et al. [34]. This is explained in an article by Favaloro et al., which demonstrated that apixaban causes DRVVC to be greater than DRVVS, resulting in a DRVVT ratio below the cutoff, unlike rivaroxaban and dabigatran, which affect DRVVS more than DRVVC resulting in a higher DRVVT ratio [21]. As shown in Tables 3, 4, true LA positive controls were falsely elevated by DOACs, but although this is not representative of the true result, the abnormal result remains abnormal.

Moreover, this study showed that DOAC-stop® and DOAC-remove® are effective DOAC-removing agents to overcome DOAC interference, resulting in accurate results. DOAC-stop® effectively removed all three DOACs below 30 ng/mL. This was also seen in the study by Favresse et al. [21]. In contrast, DOAC-remove® removed apixaban and rivaroxaban to below 30 ng/mL but not dabigatran. This trend was not observed in a study by Al-Qawzai et al., where apixaban, rivaroxaban and dabigatran were reduced to either 20 ng/mL or below with the addition of DOAC-remove®, but the median dabigatran concentration before DOAC-remove® was 66 ng/mL which was much lower than the dabigatran concentrations used in this study [35]. Removal of apixaban and rivaroxaban by DOAC-stop® and DOAC-remove® corrected all false-positive results from these DOACs, bringing them within the normal range post-treatment. However, this was not the case for dabigatran and lupus anticoagulant testing. With DOAC-stop®, all lupus anticoagulant results were brought back into range for dabigatran except DRVVS and DRVVC (at 600 ng/mL), but with DOAC-remove® only the DRVVT ratio (200 ng/mL) was brought within normal range. This can be explained by the fact that only 86% of dabigatran was removed with DOAC-remove®, compared to 99% following DOAC-stop® treatment which could be due to interference of dabigatran with the normal pool. According to the manufacturer’s instructions, a single DOAC-Remove tablet is expected to remove more than 95% of DOACs from plasma spiked with 600 ng/mL of dabigatran. However, the results indicate that this level of removal was not achieved for dabigatran, even at concentrations below 600 ng/mL. It is important to acknowledge that this study represents an experimental model and the results observed did not fully align with the manufacturer’s claims. Many articles show that DOAC-stop® and DOAC-remove® are effective in overcoming false-positive lupus anticoagulant results [16, 18–20].

Additionally, DOAC-stop® (Table 2) reduced all PTT-LA results from apixaban to below the normal range, which can be explained by the fact that the control PTT-LA for DOAC-stop® was below the pre-testing range, so after DOAC-stop® treatment, the results were lowered in conjunction with the control value. Furthermore, unlike DOAC-remove®, DOAC-stop® reduced the lupus anticoagulant results for the DOAC-spiked abnormal controls (and the controls without DOAC) more than the pre-testing control results. This resulted in the DRVVT ratio for rivaroxaban spiked abnormal controls being within the normal range, becoming false-negative. This shows that DOAC-stop® directly interfered with the abnormal controls with and without the addition of DOACs.

With dabigatran, both DOAC-stop® and DOAC-remove® were effective in bringing out-of-range protein S and ATIII (Tables 1, 2) back into the normal range and in bringing false-negative ATIII results back into the abnormal range (Tables 3, 4). In a study by Ząbczyk et al., DOAC-stop® overcame any DOAC interference that affected ATIII, which was also seen in this study [17].

Limitations of this study include the small sample size, especially in the abnormal control group. This means that certain differences observed in the study may not have reached statistical significance due to the small sample size. Therefore, further testing with a larger sample size is required. It would also be beneficial to test with actual patient samples, including patients who have antiphospholipid syndrome and thrombophilia, to determine how DOAC-stop® and DOAC-remove® interact with the samples, which avoids any interferences due to spiking the normal pool and abnormal control samples with DOACs. Additionally, although control samples were tested without the addition of DOACs it may be beneficial to carry out further testing of controls using the full procedure of adding the samples to the roller mixer and centrifuging the samples without the addition of DOAC-stop/DOAC-remove. This would help to verify the stability of the samples and ensure that there is no interference between DOAC-stop®/DOAC-remove® and the controls. A further limitation includes the adaptation of the method used to make the stock solutions using water as the solvent. According to the product information sheets of the DOACs, stock solutions could be made by dissolving the DOACs in organic solvents like dimethyl sulfoxide (DMSO). Finally, it is important to investigate whether the dilution effect of adding the DOACs influences the results of the assays. This can be achieved by adding an equivalent volume of water to normal plasma and abnormal controls which would help to determine whether the observed effects are attributable to the DOACs or if they result from dilution alone.

Overall, this study has demonstrated that patients on apixaban and rivaroxaban will have no effect on thrombophilia assays (protein S, protein C and ATIII), patients on dabigatran may cause interference with thrombophilia assays (protein S and/or ATIII) and lupus anticoagulant tests are affected by all three DOACs. This study found that both DOAC-stop® and DOAC-remove® can overcome this interference, but DOAC-stop® is better as it also overcomes interference caused by dabigatran. This study acknowledges the importance of following the British Society for Haematology (BSH) guidelines, which state that warfarin is preferred over DOACs for treating APS. Given the interference of DOACs with LA testing, waiting until DOAC therapy is completed before testing is not recommended; therefore, the use of DOAC-stop® or DOAC-remove® may be advantageous in clinical practice as they help to eliminate the effects of DOACs allowing for more accurate LA testing and avoiding unnecessary delays in initiating warfarin treatment in patients with APS.

## Summary Table

### What Is Known About This Subject?

• Although DOACs have many advantages they can interfere with laboratory assays resulting in either false-positive or false-negative results.
• DOAC removing agents such as DOAC-stop®/DOAC-remove® are effective in overcoming DOAC interference.

### What Does This Paper Add?

• Comparing the impact of apixaban, rivaroxaban and dabigatran on either normal pool plasma or abnormal controls specifically for thrombophilia and lupus testing.
• Comparison between DOAC-stop®/DOAC-remove® for overcoming DOAC interference on different assays used for thrombophilia and lupus testing.

## Concluding Statement

This work represents an advance in biomedical science because it offers a direct comparison between DOAC-stop®/DOAC-remove® for different DOACs and laboratory assays.

## Data Availability

The original contributions presented in the study are included in the article/supplementary material, further inquiries can be directed to the corresponding author.
